# Are countries’ self-reported assessments of their capacity for infectious disease control reliable? Associations among countries’ self-reported international health regulation 2005 capacity assessments and infectious disease control outcomes

**DOI:** 10.1186/s12889-020-8359-8

**Published:** 2020-03-04

**Authors:** Feng-Jen Tsai, Mathuros Tipayamongkholgul

**Affiliations:** 1 Master Program in Global Health and Development, College of Public Health, Taipei Medical University, Taipei, Taiwan; 2 PhD Program in Global Health and Health Security, College of Public Health, Taipei Medical University, Taipei, Taiwan; 3 Master of Public Health Program, Faculty of Public Health, Mahidol University, Bangkok, Thailand

**Keywords:** International health regulations (IHR), International health regulations monitoring tool (IHRMT), ProMed-mail, Self-report, Core capacity in infectious disease control

## Abstract

**Background:**

This study aimed to evaluate associations among countries’ self-reported International Health Regulation 2005 (IHR 2005) capacity assessments and infectious disease control outcomes.

**Methods:**

Countries’ self-reported assessments implemented by percentages as IHR Monitoring Tools (IHRMT) in 2016 and 2017 were used to represent national capacity regarding infectious disease control. WHO Disease Outbreak News and matched diseases reports on ProMED-mail were collected in 2016 to represent disease control outcomes of countries. Disease control outcomes were divided in good, normal and bad groups based on the development of outbreaks listed in the reports. The Human Development Index (HDI), density of physicians and nurses, health expenditure, number of arrivals of international tourists were also collected for control. Chi-square test and logistic regression were applied for analysis.

**Results:**

A total of 907 cases occurred in 92 countries. For all diseases, cases occurring in high international travel volume countries presented twice the risk of having a bad disease control outcomes than cases occurring in low international travel volume countries (OR = 2.19 for IHR 2016, OR = 2.97 for IHR 2017). Cases occurring in low IHR average score countries had significant higher risk (OR = 7.83 for IHR 2016 and OR = 2.23 for IHR 2017) of having a bad disease control outcomes than countries with high IHR average scores. For only human diseases, cases occurring in high international travel volume countries presented twice the risk of having a bad disease control outcomes than cases occurring in low international travel volume countries for IHR 2017 (OR = 2.79). Cases occurring in low IHR average score countries had significant higher risk (OR = 11.16 for IHR 2016 and OR = 3.45 for IHR 2017) of having a bad disease control outcomes than countries with high IHR average scores. The HDI, health workforce density and total health expenditure were all positively associated with disease control outcomes.

**Conclusions:**

Countries’ self-reported infectious disease control capacities positively correlated with their disease control outcomes. While the self-reported IHR scores were accountable to some degree, this approach was useful for understanding global capacity in infectious disease control and in allocating resources for future preparedness.

## Background

Infectious diseases are one of the most significant health and security challenges for the world damaging global economics and public health [1–3]. After the SARS pandemic in 2003, International Health Regulations 2005 (IHR 2005) were adopted by the World Health Organization (WHO) to enhance the global capacity to prevent and control infectious diseases [4]. One of the approaches adopted by IHR 2005 is to require member states to develop minimal core public health capacities to implement the IHR 2005 effectively.

To monitor progress in this regard, WHO introduced a self-assessment process for countries to report on their implementation of IHR 2005 [5]. The IHR Secretariat at WHO developed the IHR Core Capacity Monitoring Framework and released the IHR Monitoring Tool (IHRMT) to monitor progress in implementing IHR core capacities in 2010 [6]. With this standardized data collection tool, countries were required to fill out the IHRMT and submit completed reports to WHO annually [7].

This self-report process received such insufficient attention that in 2014, only 60 countries reported their self-assessment to WHO. The failures concerning the 2014 to 2016 Ebola outbreak in West Africa have resulted in a multitude of review panels, many of which agreed that the self-assessment process was flawed - in that it did not necessarily reflect an accurate picture of national capacity for disease control [8–10]. With this weakness, the review panels recommended a shift of mechanisms from the self-report to Joint External Evaluations (JEE) concerning national capacities in pandemic preparedness [11].

Self-reports are a widely used approach in collecting health related information from individuals. Studies on health behavior have revealed the weakness of the self-report approach, for example, being subject to relativism, and influenced by engagement and culture [12–14]. Similarly, studies on organizational behavior in health promotion have uncovered the bias of self-reports including under-reporting inappropriate behaviors and over-reporting appropriate behavior [15]. This tendency to respond in socially desirable ways has created a problem involving information accuracy. When the WHO uses IHR self-reported information to allocate resources to strengthen national capacity in infectious disease control, this might mislead the focus if the information is inaccurate [16]. Although external evaluation could partly solve the problem, understanding the reliability of national self-reported capacities is still important because it remains the main approach in collecting information from countries. However studies have rarely focused on the accuracy of national self-reported information.

To explore whether and to what extent the self-reported approach reflect countries’ real capacities for infectious disease control, we conducted the study with the hypothesis that countries’ self-reported IHR capacity would correlate to their infectious disease control outcomes.

## Methods

### IHR self-reported capacity

IHRMT is a questionnaire to monitor progress in implementing the IHR of countries [5]. The questionnaire consists of 13 sections including 8 core capacities, points of entry and 4 ‘other hazards’ as identified and delineated by the WHO to match the obligations outlined in Annex 1 of the IHR. Eight core capacities mainly for infectious disease control include legislation, coordination, surveillance, response, preparedness, risk communication, human resources and laboratory. The 4 hazards include zoonosis, food safety, chemical and radionuclear. Individual questions were grouped by components and indicators in the questionnaires including 256 total attributes.

The response for IHRMT from countries comprises the percentage of implementation ranging from 0 to 100. We obtained countries’ self-reported implementation percentages as scores from the WHO website on 31rd October 2018 [17]. One hundred countries’ self-reported IHRMT scores in 2016 were available and used in the study. While there are more countries (*n* = 160) reported IHR scores in 2017, we also collected self-reported IHRMT in 2017 for analysis. The average score of 8 core capacities was further calculated to represent overall national capacity regarding infectious disease control.

### Infectious disease control outcomes

Based on the rationale that early detection and effective response to avoid further level up the pandemic is fundamental in infectious disease control, we use the report information from ProMED-mail and WHO Disease Outbreak News to be the indicator of infectious disease control outcome because both systems aim at early reporting of the outbreak and updated the development of the possible pandemic.

To evaluate infectious disease control outcomes, we first collected all disease outbreak reports in 2016 released on the WHO Disease Outbreak News website [18]. Also, we collected all WHO outbreak reports concerning diseases, i.e., avian flu, yellow fever, and Middle East respiratory syndrome and coronavirus (MERS-CoV), those having been reported on the WHO website- from ProMED-mail in 2016. ProMED-mail is a nongovernmental emerging disease monitoring program established in 1994 to provide early warning about outbreaks based on information from various sources [19]. The credibility of ProMED-mail and its efforts on reporting timely information were repeatedly confirmed by several studies [20–22]. By collecting all sources of information including media reports, official reports, online summaries, local observers, and others without political constraints, reports on ProMED-mail is comprehensive. As an internet-based reporting system with electronic communications approach, the effect of reporting in a timely manner of ProMED-mail was also confirmed by previous study through comparing the timeliness of reporting form the WHO. Apart from the WHO, which reports “a public health emergency of international concern” regulated by IHR 2005, ProMED-mail aims at reporting all kinds of information on outbreaks of infectious diseases. Thus, we collected outbreak information from both websites to track countries’ infectious disease control situations. Reports containing only animal disease outbreaks were also collected.

As for multiple countries outbreak reports from WHO and ProMED-mail, each country report was separated as an individual case. Aside from initial outbreak reports, WHO Disease Outbreak News also posts reports labeled as “update”. These reports were examined for details indicating the spread of the initial outbreak to other regions in the affected countries. We searched ProMED-mail reports to match the information about the spread of outbreaks to other countries. Updates that mentioned only an increased number of cases without additional information about geographical spread within the country were excluded. Reports about WHO technical meetings and epidemiological survey findings were also excluded. Then we matched the outbreak reports of WHO and ProMED-mail based on the information revealed in the report including disease name, country and the date of onset and other details.

After matching, we ranked the infectious disease control outcomes of reports based on the rationale that the spread of infectious diseases was controlled right after their detection, and might represent better control outcomes of the country. Disease control outcomes were ranked in 4 levels. Reports containing only animal cases were ranked as level 1. Human disease reports which were only listed on ProMED-mail were ranked as level 2. Human disease outbreaks updated in ProMED-mail showing the spread of disease to other regions of the country were ranked as level 3. Lastly, the disease outbreaks listed on both ProMED-mail and the WHO website or only listed on the WHO website were ranked as level 4 (the worst), meaning that disease was out of control and had become a global concern.

We collected the earliest 10 cases from each rank to be the subset for a validation of ranking methodology. Two researchers individually ranked the cases into 4 levels based on the review of the outbreak information including case count (died, confirmed and suspected cases), spread, or other related indicators provided in the report. The agreement rate among these two researchers was 90%. And the average ranking level was parallel with the original ranking level.

Using this method, 907 reports were collected to analyze.

### Measurements

With the rationale that national infectious disease control capacity includes systematic elements like legislation and coordination and human resources as trained medical professionals [11, 23], we further searched the Human Development Index (HDI) from the United Nations Development Program (UNDP) and information from WHO regarding the density of physician and nurses and total health expenditure to represent the general health capacity of the country [2, 24].

Human development is defined as encompassing three dimensions: life expectancy at birth as an index of population health and longevity; knowledge and education as measured by the adult literacy rate and the combined primary, secondary and tertiary gross enrollment ratio and standard of living as measured by the natural logarithm of gross domestic product per capita at purchasing power parity. With indicators mainly collected from official statistics, the indexes of the three dimensions were expressed as a value between 0 and 1 by applying the general formula. Then the human development index was calculated as a simple average of the dimension indices ranging between 0 and 1, with 1 representing the highest degree of human development and 0 the lowest. We used the human development index of 2016 to represent the human development status of each country in that year. The details of methods to determine the values are described in the Technical Notes section of the report [24]. In addition, the categories used by the UN, i.e., very high, high, medium and low development countries were also used in the study.

Information of each country’s density of physicians and nurses was collected from WHO websites [2]. Then the sum of these two scores was calculated and used as the index of the health workforce in the study. We then categorized countries as having a high, middle or low health workforce according to the sum of the density of physicians and nurses in each country. Countries with upper tertile scores of health workforce density were defined as having a high health workforce. Countries with the middle and lower tertile scores of health workforce density were defined as having a middle and low health workforce, respectively.

Information of each country’s total health expenditure was also collected from WHO websites to represent the national investment in health. We then categorized countries into three groups: countries with upper tertile scores was defined as having a high total health expenditure, the others were defined as having a middle and low total health expenditure, respectively.

While the frequency of international travel increases the risk of infectious disease outbreak, we also collected information regarding the number of arrivals of international tourists from the World Bank to represent the risk of exposure to infectious diseases [4]. The World Bank classifies the number of arrivals of international tourists in 10 levels. We reclassified countries in 2 international travel groups (high vs. low) using the cut-off point at level 5.

### Data analysis

IHR average score was categorized as high, middle or low. Countries with upper tertile scores (≧97.6) were defined as having a high IHR average score. Countries with middle (88.89 to 97.5) and lower tertile scores (≦88.88) were defined as having middle and low IHR average scores, respectively. While the upper tertile point of IHR average score of 2017 was 99.25, we divided the scores into two levels, high vs. low, using the group mean (86.105) as the cut-off point to avoid the bias of excessive concentration.

Reports were further divided by disease control outcomes in 3 groups. Reports with a disease control level 1 and level 2 were classified as “good”. Reports with a disease control level 3 and 4 were classified as “normal” or “International alert or bad”.

Chi-square test was applied to compare differences among HDI, health workforce, international travel, total health expenditure and IHR self-reported scores among diverse disease control outcome groups. Then reports with normal or bad disease control outcomes were combined and analyzed further. Logistic regression was then adopted to estimate the associations among disease control outcomes and IHR self-reporting scores, HDI, health workforce and international travel. Two models were applied in the analysis where the regression was used for all cases and for only human cases separately.

All analysis was performed using the software SPSS, Version 18.0.

## Results

### IHR self-reported scores

Scores of IHR core capacities reported by country are shown in Table 1. The 907 data we collected were reported in 97 countries. Among 72 countries (727 reports) had IHR scores in 2016, the average score of all indicators ranged from 75.56 to 94.42. Among 84 countries (863 reports) had IHR scores in 2017, the average score of all indicators ranged from 68.23 to 91.95. For both IHR average scores of 2016 and 2017, zoonosis (94.42 in 2016 and 91.95 in 2017) and surveillance (93.55 in 2016 and 90.28 in 2017) were both within the top 3 capacities with high scores while points of entry (75.56 in 2016 and 68.23 in 2017) exhibited the lowest capacity score. Using standard deviation, points of entry and legislation were the top two capacities with the greatest gap among countries both in 2016 and 2017. And the most homogeneous capacity among countries was surveillance and zoonosis.

**Table 1:** Scores of IHR core capacities reported by country in 2016 and 2017

	Self-reported IHR Scores							
	2016 (N of countries = 72)				2017 (N of countries = 84)			
	Mean	SD	Range	N of country scored 100	Mean	SD	Range	N of country scored 100
IHR core capacitites								
Legislation	84.61	29.36	0~100	45	85.31	28.10	0~100	48
Coordination	91.88	13.84	20~100	37	90.18	19.80	0~100	45
Surveillance	93.55	10.5	30~100	26	90.28	12.09	25~100	20
Response	88.88	20.11	0~100	29	86.10	23.59	6~100	25
Preparedness	87.59	21.75	0~100	29	83.00	27.33	0~100	32
Risk communication	90.01	18.12	14~100	39	86.31	24.55	14~100	39
Human resources	80.61	24.94	0~100	31	77.31	26.42	0~100	34
Laboratory	85.44	18.62	39~100	24	90.35	17.31	17~100	27
Points of entry	75.56	34.9	0~100	12	68.23	37.70	0~100	11
Zoonosis	94.42	12.86	33~100	49	91.95	15.45	22~100	49
Food safety	83.16	18.57	0~100	30	85.21	22.30	0~100	36
Average score of 8 core capacities*	87.82	15.46	35.38~100	8	86.11	19.69	24.13~100	6

* 8 core capacities included legislation, coordination, surveillance, response, preparedness, risk communication, human resources and laboratory

In addition, zoonosis was the indicator reported as 100 by most of the countries both in 2016 and 2017 (*n* = 49), followed by legislation (*n* = 45 in 2016, *n* = 48 in 2017).

Compared with IHR 2016, IHR average scores of 2017 is 1.26 point lower. And the average score of all indicators in 2017 were lower than scores in 2016.

### Comparison of HDI, health workforce, international travel, total health expenditure and IHR scores among disease control outcome groups using chi-square

Comparison of HDI, health workforce, international travel, total health expenditure and IHR scores among disease control outcome groups using Chi-square were showed in Table 2. Among all reports, 227 reports concerned avian flu (25%), 152 studied yellow fever (16.8%) and 142 examined Middle East respiratory syndrome coronavirus (MERS-CoV, 15.7%) reports. As for human reports, 186 studies examined avian flu (23.3%), 144 studied yellow fever (18%), and 135 considered MERs-CoV (16.9%) reports.

**Table 2:** Comparisons of factors between different disease control status

	Disease control status													
	All cases^a^ (***N*** = 907)							Only human cases (***N*** = 800)						
	Good^b^		Normal		International alert or bad			Good		Normal		International alert or bad		
	***n*** = 492		***n*** = 148		***n*** = 267			***n*** = 385		n = 148		n = 267		
	n	%	n	%	n	%	Chi-square	n	%	n	%	n	%	Chi-square
HDI														
Very High	276	56.10	52	35.14	103	38.58	139.23***	222	57.66	52	35.14	103	38.58	137.183***
High	69	14.02	6	4.05	93	34.83		46	11.95	6	4.05	93	34.83	
Middle-low	67	13.62	18	12.16	26	9.74		50	12.99	18	12.16	26	9.74	
low	79	16.06	72	48.65	45	16.85		66	17.14	72	48.65	45	16.85	
International travel volume														
Low	48	9.76	59	39.86	62	23.22	72.34***	39	10.13	59	39.86	62	23.22	60.951***
High	436	88.62	88	59.46	203	76.03		340	88.31	88	59.46	203	76.03	
Health workforce density														
High	183	37.20	16	10.81	65	24.34	142.42***	140	36.36	57	10.81	96	24.34	132.357***
Middle	81	16.46	4	2.70	101	37.83		57	14.81	4	2.70	101	37.83	
Low	110	22.36	82	55.41	68	25.47		96	24.94	82	55.41	68	25.47	
Total Health Expenditure														
High	183	37.20	62	41.89	40	14.98	172.00***	120	31.17	62	41.89	40	14.98	119.312***
Middle	159	32.32	4	2.70	143	53.56		146	37.92	4	2.70	143	53.56	
Low	140	28.46	81	54.73	74	27.72		114	29.61	81	54.73	74	27.72	
IHR average scores														
2016														
High	164	33.33	3	2.03	55	20.60	84.88***	141	36.62	3	2.03	55	20.60	84.45***
Middle	106	21.54	52	35.14	103	38.58		74	19.22	52	35.14	103	38.58	
Low	149	30.28	42	28.38	52	19.48		123	31.95	42	28.38	52	19.48	
2017														
High	313	63.62	109	73.65	192	71.91	24.197***	234	60.78	109	73.65	192	71.91	15.177**
Low	153	31.10	36	24.32	60	22.47		138	35.84	36	24.32	60	22.47	

^a^ including animal and human cases

^b^ good included infectious control level 1 and level 2

*p* < 0.1*, *p* < 0.05** *p* < 0.01***

For all cases, HDI, international travel, health workforce, total health expenditure and IHR average scores all significantly differed among disease control outcome groups. In the good disease control outcome group, cases frequently occurred in very high HDI (56%), high international travel volume (88%), high health workforce (37.20%) and high health expenditure (37.20%) countries. In the normal disease control outcome group, cases often occurred in high international travel volume (59.46%) but low HDI (48.65%), low health workforce (55.41%) and low total health expenditure (54.73%) countries. Concerning the bad disease control outcome group, cases usually occurred in very and high HDI (38.58 and 34.83%), high international travel volume (76.03%) but middle health workforce (37.83%) and middle total health expenditure (53.56%) countries.

Regarding IHR self-reported scores, 33.33% of cases in the good disease control outcome group occurred in countries with high IHR average scores in 2016 while 35.14% cases were found in the normal group and 38.58% cases in the bad group occurred in middle IHR average scores countries. For IHR self-reported scores in 2017, 31.10% of cases in the good disease control outcome group occurred in countries with low IHR average scores while 24.32% in normal group and 22.47 in the bad group occurred in low IHR average scores countries.

Similarly, HDI, international travel, health workforce, total health expenditure and IHR average scores both in 2016 and 2017 all significantly differed among disease control outcome groups for only human case analysis. In the good disease control outcome group, cases frequently occurred in very high HDI (57.66%), high international travel volume (88.31%), high health workforce (36.36%) but middle total health expenditure (37.92%) countries. Regarding IHR self-reported scores, 36.62% of cases in the good disease control outcome group occurred in countries with high IHR average scores in 2016 while 35.14% cases were found in the normal group and 38.58% cases in the bad group occurred in middle IHR average scores countries. For IHR self-reported scores in 2017, 35.84% of cases in the good disease control outcome group occurred in countries with low IHR average scores while 24.32% in normal group and 22.47 in the bad group occurred in low IHR average scores countries.

### Associations between HDI, health workforce, international travel, IHR scores and disease control outcomes revealed by binary logistic regression

Associations between HDI, health workforce, international travel, IHR scores and disease control outcomes are shown in Table 3. Regarding analysis with IHR score in 2016 for all cases, HDI, international travel, total health expenditure and IHR average scores were significantly associated with disease control outcomes. Cases occurring in high HDI (OR = 2.23) and low HDI countries had higher risk (OR = 1.84) of having bad disease control outcomes than very high HDI countries. Cases occurring in high international travel volume countries had twice the risk of having bad disease control outcomes than cases occurring in low international travel volume countries (OR = 2.19). Cases occurring in low total health expenditure countries had nearly four times risk of having bad disease control outcomes than countries with high health expenditure (OR = 3.99). And cases occurring in low IHR average scores countries had 5 times the risk (OR = 7.83) of having bad disease control outcomes than in countries with high IHR average scores.

**Table 3:** Associations between disease control status and HDI, travel amount, health workforce and IHR scores

	Risk of bad Disease control status			
	2016		2017	
	All cases	Only human cases	All cases	Only human cases
	OR (95% CI)	OR (95% CI)	OR (95% CI)	OR (95% CI)
HDI				
Very High	1	1	1	1
High	2.23 (1.15–4.35)*	1.84 (0.91–3.74)	4.71 (2.53–8.75)***	3.83 (2.00–7.33)***
Middle-low	1.97 (1.00–3.89)	2.65 (1.25–5.63)*	2.29 (1.24–4.21)**	2.96 (1.53–5.75)**
low	1.84 (1.04–3.27)*	1.68 (0.87–3.25)	3.59 (2.13–6.07)***	3.11 (1.75–5.54)***
International travel volume				
Low	1	1	1	1
High	2.19 (1.15–4.19)*	1.85 (0.95–3.60)	2.97 (1.68–5.25)***	2.79 (1.51–5.18)**
Health workforce density				
High	1	1	1	1
Middle	0.72 (0.27–1.92)	0.60 (0.19–1.84)	2.59 (1.07–6.31)*	3.17 (1.18–8.54)*
Low	0.80 (0.41–1.56)	0.73 (0.34–1.55)	1.55 (0.88–2.74)	2.24 (1.19–4.22)*
Total Health Expenditure				
High	1	1	1	1
Middle	2.08 (0.96–4.52)	1.78 (0.82–3.88)	0.83 (0.43–1.59)	0.75 (0.38–1.49)
Low	3.99 (2.18–7.29)***	2.84 (1.51–5.35)**	2.79 (1.68–4.61)***	1.85 (1.08–3.17)*
IHR average scores				
High	1	1	1	1
Middle	1.85 (0.89–3.85)	2.05 (0.86–4.89)	NA	
Low	7.83 (4.10–14.95)***	11.16 (5.27–23.61)***	2.23 (1.25–3.96)**	3.45 (1.79–6.66)***

*p* < 0.1*, *p* < 0.05** *p* < 0.01***

For only human cases, associations among HDI, total health expenditure and IHR average scores in 2016 and disease control outcomes were statistically significant. Cases occurring in middle to low HDI countries had twice as high a risk of having bad disease control outcomes than those in very high HDI countries (OR = 2.65). Cases occurring in low total health expenditure countries had two times risk of having bad disease control outcome than countries with high health expenditure (OR = 2.84). Cases occurring in low IHR average scores countries had an 11 times higher risk (OR = 11.16) of having bad disease control outcomes than countries with high IHR average scores.

Regarding analysis with IHR score in 2017 for all cases, HDI, international travel, health workforce density, total health expenditure and IHR average scores were all significantly associated with disease control outcomes. Cases occurring in high HDI (OR = 4.71), middle-low HDI (OR = 2.29) and low HDI countries had higher risk (OR = 3.59) of having bad disease control outcomes than very high HDI countries. Cases occurring in high international travel volume countries had twice the risk of having bad disease control outcomes than cases occurring in low international travel volume countries (OR = 2.97). Cases occurring in middle health workforce density countries had two times risk of having bad disease outcomes than countries with high health workforce countries (OR = 2.59). Cases occurring in low total health expenditure countries had two times risk of having bad disease control outcomes than countries with high health expenditure (OR = 2.79). And cases occurring in low IHR average scores countries had 2 times the risk (OR = 2.23) of having bad disease control outcomes than in countries with high IHR average scores.

Similarly, for only human cases, associations among HDI, international travel, health workforce density, total health expenditure and IHR average scores and disease control outcomes were all statistically significant. Cases occurring in low IHR average scores countries had 3 times the risk (OR = 3.45) of having bad disease control outcomes than in countries with high IHR average scores.

The Odds Ratio of IHR 2017 is lower than the Odds Ratio of IHR 2016.

## Discussion

To our knowledge, this is the first study evaluating the accuracy of countries’ self-reported infectious disease control capacities. Our study results a positively correlated between countries’ self-reported IHR average scores and disease control outcomes. It suggested that the higher the IHR self-reported scores were, the better the disease control outcomes the countries had. Although some countries reported a score of 100 for all items, which was unreasonable, as a whole, countries’ self-reported IHR scores could predict their disease control outcomes.

Generally, countries tended to report high scores in all indicators. Therefore the average scores for all IHR indicators were higher than 80 in both 2016 and 2017, except for points of entry and human resources in 2017. Fortunately, on average, countries reported scores with minor partial adjustments showing their real capacities. While countries’ self-reported IHR scores were accountable to some degrees, this self-reported approach was useful in contributing to the world’s body of knowledge regarding the whole picture of national capacity concerning infectious disease control. Also, it would be useful regarding resource allocations to strengthen infectious disease control capacity.

From the study results, countries with low IHR average scores of 2016 presented an 11 times higher risk of having bad infectious disease outcomes concerning human cases and a 7 times higher risk of having bad infectious disease outcomes for all cases than countries with high IHR average scores. Countries with low IHR average scores of 2017 presented an 2 times higher risk of having bad infectious disease outcomes concerning human cases and a 3 times higher risk of having bad infectious disease outcomes for all cases than countries with high IHR average scores. This fact indicates the emerging need to examine low IHR score countries more closely to avoid the loopholes of global infectious disease control. Especially, middle to low HDI countries with low health workforce density, low total health expenditure and high international travel volume should constitute a priority.

The finding that countries with high international travel volumes had a twice as high risk of bad disease control outcomes might suggest a lack of capacity in those countries in handling high amounts of international travelers, especially travelers with animals or food products. While controlling the spread of human infection is already difficult, these countries might lack the capacity to monitor and control cases of bringing animals and food in the airport. The finding that the point of entry indicator received the lowest scores might be one support for this finding.

The finding that countries with low health workforce density and low total health expenditure had significantly higher risk in having bad disease control outcomes represented the lack of health resources of countries to respond to the request of IHR. The comparatively low scores of human resources might be the support for this finding.

Interestingly, the average scores of IHR was decreased from 87.82 in 2016 to 86.11 in 2017. Also, the Odds Ratio of IHR 2017 is lower than the Odds Ration of IHR 2016. One of the possible explanations of the phenomenon might be the impact of JEE. While there is objective external review for national capacity, countries might adjust their evaluation in 2017. So the IHR scores in 2017 is lower and the ORs of IHR data in 2017 is better than IHR data in 2016. Further study is needed to understand the reason for this change.

The current development of strengthening national core capacity of infectious disease control is to encourage countries to go through external evaluation. Based on the study outcome, we recommend the combination of external and self-report approach in the future. The stress of external evaluation had the effect of justifying the self-evaluation outcomes. But frequent external evaluation is not necessary. Instead, close monitor of countries’ self-reported outcome is highly recommended in order to understand the status and change of countries’ core capacity for better global governance in infectious disease control. In addition, the study finding might generalize to other field of global evaluations like capacity for animal and plant health inspection in agriculture sector due to the similarity of self-reporting process of the country.

Several limitations were noted in the study. First, we could not rule out the possible blockade and control of information for disease in some countries, so possibly their capacities were overvalued in the study. Second, a relationship could only be considered associations rather than causal due to the cross-sectional design. Third, three diseases, i.e., avian flu, yellow fever, and MERS-CoV, together accounted for over 50% of the reports and outbreak reports were concentrated in specific countries. Therefore, the performance observed in these affected countries had a great impact on overall outcomes. The WHO sometimes reported eye-catching cases although it constituted just one case in one country. We might have under-rated the disease control outcomes in such cases, though rarely. Forth, countries’ self-reported assessments were not validated. However, this is the reason why we conducted this study to see the reliability of such approach and information. Fifth, our infectious disease control outcomes are measured by the level of pandemic only and other indicators such as prevalence, incidence and mortality are not taken into account. Further comprehensive study is recommended.

## Conclusions

In conclusion, we found a positive correlation between countries’ self-reported infectious disease control capacities evaluated by IHRMT and their disease control outcomes. While self-reported IHR scores were accountable to some degree, frequent monitor of self-report and regular external evaluation are recommended for the future for understanding national capacity in infectious disease control and in allocating resources for global infectious disease preparedness.

## Data Availability

The datasets used and/or analysed during the current study are available from the corresponding author on reasonable request.

## Abbreviations

HDI: Human development index
IHR 2005: International Health Regulations 2005
IHRMT: IHR Monitoring tools
JEE: Joint external evaluations
MERS-CoV: Middle East respiratory syndrome and coronavirus
OR: Odds ratio
UNDP: United Nations Development Program
WHO: World Health Organization
